# Ascending aortic aneurysm in a patient with bicuspid aortic valve, positive history of systemic autoimmune diseases and common genetic factors: a case report

**DOI:** 10.1186/1476-7120-7-34

**Published:** 2009-07-06

**Authors:** Ilenia Foffa, Pier Luigi Festa, Lamia Ait-Ali, Annamaria Mazzone, Stefano Bevilacqua, Maria Grazia Andreassi

**Affiliations:** 1 Institute of Clinical Physiology, Massa, Italy; 2 The Sant'Anna School of Advanced Studies, Pisa, Italy; 3 G. Monasterio Foundation, CNR-Regione Toscana, G: Pasquinucci Hospital, Massa, Italy

## Abstract

The bicuspid aortic valve (BAV) and specific systemic autoimmune diseases are associated with cardiovascular manifestation, including aortic aneurysm. We reported a case of 64 year-old patient with BAV and a history of ankylosing spondylitis (AS) and systemic lupus erythematosus (SLE), and who developed ascending thoracic aortic aneurysm. The patient presented also the homozygosity for genetic variants of MMP9, ACE, MTHFR and PAI-1 genes. Gene-environmental interactions may represent an additional pathogenetic dimension in the still challenging management of the abnormalities of the aortic wall, including dilatation, aneurysm and dissection.

## Introduction

The bicuspid aortic valve (BAV) affects 1 to 2% of the population and may be complicated by abnormalities of the aortic wall, including dilatation, aneurysm and dissection [1].

Specific systemic autoimmune diseases are associated with cardiovascular manifestations, including aortic aneurysm [2,3].

We reported a case of a 64-year-old male with BAV and a history of ankylosing spondylitis (AS) and systemic lupus erythematosus (SLE), and who developed ascending thoracic aortic aneurysm. In addition we have performed a genetic screnning of 5 gene polymorphisms (ACE I/D, MTHFR 677C>T, MMP9-1562C>T, PAI 1 4G/5G; MMP12 A82G) reported in multiple case control studies to be genetic risk factors for the development of the abdominal aortic aneurysm (AAA) [4].

## Case presentation

The patient was a -64 year-old man, who had a 25-year history of hypertension and a 8-year history of hypothyroidism. In 2000, the patient underwent a corrective osteotomy of the spine for ankylosing spondylitis and spondylarthropathy. He had also been diagnosed as having a dilatation of the ascending thoracic aorta associated to BAV. The patients was treated with metilprednisolone along with colchicine, and was followed regularly for progressive aortic dilation.

**Figure 1:**
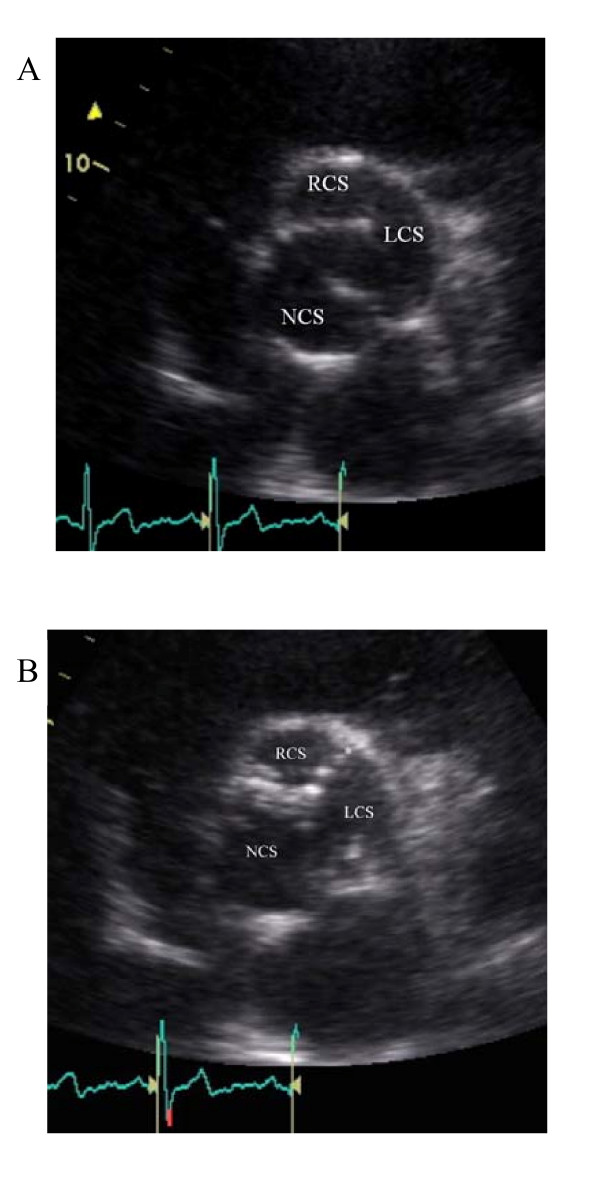
Parasternal short axis view of aortic valve during systole (A) and diastole (B): BAV with "rafe" (*) between the left coronary sinus (LCS) and right coronary sinus (RCS). NCS: non coronary sinus.

**Figure 2:**
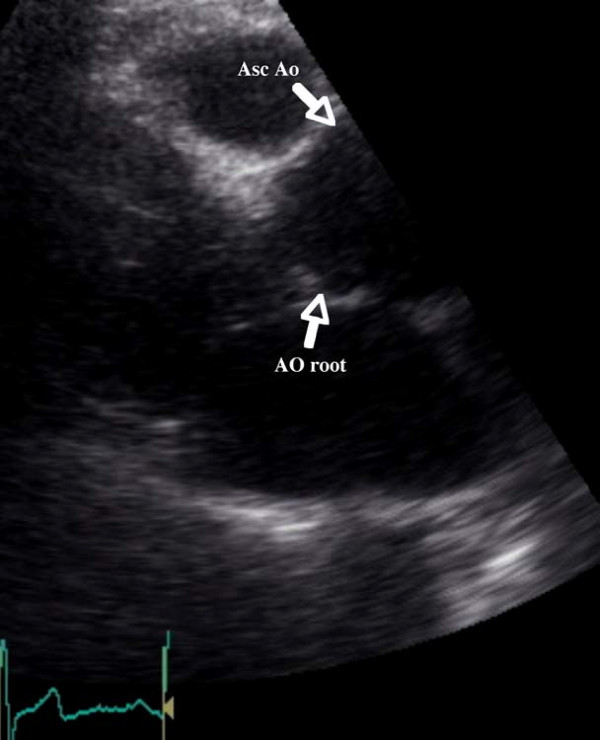
**Parasternal long-axis view showing a dilated aortic root and ascending aorta**. Ao: Aorta; Asc Ao: Ascending aorta.

He was admitted at our hospital for further evaluation on December 2008. Transthoracic echocardiography revealed aortic root and ascending aortic dilatation (Figures 1, 2).

A computed tomography scan confirmed the presence of the ascending aorta aneurysm (53 mm) and ectasia of the aortic root and arch (respectively 45 mm and 42 mm). The patient was referred to our cardiothoracic surgery service in order to evaluate the need for aortic root replacement. Preoperative coronary angiography and aortography confirmed the presence of ascending aortic aneurysm root without evidence of hemodynamically significant stenosis or regurgitation so the cardiac surgeons decided to postpone surgical treatment at the time when the valve requires replacement.

Genetic testing was performed by polymerase chain reaction/restriction fragment length polymorphism analysis in order to identify genotypes patient. The genetic results for the studied polymorphisms are shown in table 1. The patient was TT, DD, 5G5G and TT homozygous for the MMP9-1562C, ACE I/D, PAI 1 4G/5G and MTHFR C677T genetic polymorphisms, respectively.

**Table 1:** Genotypes of the studied candidate genes

**Gene**	**Polymorphism**	**Biological function**	**Genotypes**
MTHFR	C677T	Reduction in enzyme activity and moderate increase in plasma homocysteine levels	TT

MMP9	C1562T	~2-fold higher promoter activity	TT

MMP12	A82G	Different transcriptional activity (upregulation)	AA

ACE	I/D	I allele is associated with 50% decrease in ACE levels	DD

PAI-1	4G/5G	The 5G variant is associated with less inhibition of the plasminogen activators and, consequently, increased conversion of plasminogen to plasmin and increased activation of MMPs.	5G/5G

## Discussion

Patients with AS and SLE may develop cardiovascular manifestations ranging from asymptomatic forms to life threatening conditions, including common cardiovascular manifestation and valvular problems [2,3]. The mechanism responsible for the occurrence and progression of aortic dilatation has also not yet been elucidated in detail. Aortic aneurysm may be the result of the medial degeneration, induced by chronic inflammation and accelerated by prolonged corticosteroid therapy.

Aneurism formation may be more common in patients with the coexistence of BAV. In fact, bicuspid aortic valve is considered to be a cause of intrinsic changes in the aortic wall resulting in aneurysms of the ascending aorta, independent of degree of valvular dysfunction [1].

In addition to the presence of BAV and the inflammatory involvement of the aortic wall by immune diseases and systemic hypertension, genetic factors may contribute significantly to the development of aortic dilation in our patient. Indeed, our observations are in agreement with such hypothesis, reporting the presence of homozygosity for genetic variants of MMP9, ACE, MTHFR and PAI-1 genes that have been previously associated with a significant risk of abdominal aortic aneurysm disease [4]. Expression of MMP-9 is elevated in vascular disease, and in particular within aneurysm tissues. A meta-analysis of 2 larger case-control studies that have looked at the ACE I/D polymorphism in AAA patients showed a strong overall association between ACE D allele (RR 1.33 [1.20e1.48]) and disease [4].

An association between the presence of AAA and elevated MTHFR 677C>T has been indicated, and meta-analysis of these studies reveal a significant increased risk of AAA disease for the T allele variant [4]. Recently, a study suggested a significant association between growth and plasminogen activator inhibitor (PAI) 1–675 4G/5G and AAA [5].

In the present case report, in addition to inflammatory involvement of the aortic wall by systemic autoimmune diseases, these specific genetic variants may have promoted the development of aortic dilation in this patient.

BAV is responsible for a large proportion of patients coming to aortic valve replacement. The mechanism responsible for the associated vascular complications remains controversial. Some patients with BAV have rapidly progressive valve and aortic dysfunction while some remain without complications.

Several advances in the molecular genetics of aortic valve disease related to BAV, have recently been made, especially through the use of linkage analysis. These resulted in the discovery of mutations in NOTCH1 gene, a signaling and transcriptional regulator gene on chromosome 9, NOTCH1 and some different loci linked to BAV on chromosomes 18, 5, and 13 [6].

However, genetic basis of BAV remains unclear. Our data suggest, for the first time, that some genetic biomarkers (functional polymorphisms) may predispose BAV patients at an increased risk of aortic dilatation, aneurysm formation, and dissection. Our findings are hypothesis generating and need to be confirmed by further clinical studies.

Elucidating the genetic basis for BAV may have substantial implications in clinical practice. Identification of specific genetic markers may helpful for early clinical detection of relatives; genetic markers might be also used to predict the natural progression of the condition and to identify those cases that might have potentially life-threatening complications from BAV

Therefore, future studies focusing on the identification of additional disease-causing and susceptibility genes are needed in order to improve understanding of the pathophysiological processes as well as to identify new therapeutic strategies.

## Consent

Written informed consent was obtained from the patient for publication of this case report and any accompanying images. A copy of the written consent is available for review by the Editor-in-Chief of this journal

## Competing interests

The authors declare that they have no competing interests.

## Authors' contributions

IF carried out the molecular genetic studies and helped to draft the manuscript; LA and PLF have been involved in revising it critically for important intellectual content; AM performed the echocardiography; SB give his sugery opinion and has been involved in revising of the manuscript; MGA defined the study design and wrote the manuscript.

All authors read and approved the final manuscript.
